# Transcriptional and Epigenetic Bioinformatic Analysis of Claudin-9 Regulation in Gastric Cancer

**DOI:** 10.1155/2021/5936905

**Published:** 2021-12-18

**Authors:** Elizabeth Hernández-Nava, Luis F. Montaño, Erika P. Rendón-Huerta

**Affiliations:** Laboratorio Inmunobiología, Departamento Biología Celular y Tisular, Facultad de Medicina, UNAM, Mexico City, Mexico

## Abstract

Gastric cancer is a heterogeneous disease that represents 5% to 10% of all new cancer cases worldwide. Advances in histological diagnosis and the discovery of new genes have admitted new genomic classifications. Nevertheless, the bioinformatic analysis of gastric cancer databases has favored the detection of specific differentially expressed genes with biological significance. Claudins, a family of proteins involved in tight junction physiology, have emerged as the key regulators of cellular processes, such as growth, proliferation, and migration, associated with cancer progression. The expression of Claudin-9 in the gastric cancer tissue has been linked to poor prognosis, however, its transcriptional and epigenetic regulations demand a more comprehensive analysis. Using the neural network promoter prediction, TransFact, Uniprot-KB, Expasy-SOPMA, protein data bank, proteomics DB, Interpro, BioGRID, String, and the FASTA protein sequence databases and software, we found the following: (1) the promoter sequence has an unconventional structure, including different transcriptional regulation elements distributed throughout it, (2) GATA 4, GATA 6, and KLF5 are the key regulators of Claudin-9 expression, (3) Oct1, NF-*κ*B, AP-1, c-Ets-1, and HNF-3*β* have the higher binding affinity to the CLDN9 promoter, (4) Claudin-9 interacts with cell differentiation and development proteins, (5) CLDN9 is highly methylated, and (6) Claudin-9 expression is associated with poor survival. In conclusion, Claudin-9 is a protein that should be considered a diagnostic marker as its gene promoter region binds to the transcription factors associated with the deregulation of cell control, enhanced cell proliferation, and metastasis.

## 1. Introduction

Gastric cancer is the third leading cause of cancer-related deaths worldwide [1]. There are two main subtypes of gastric cancer, namely intestinal and diffuse. The intestinal type is associated with *H. pylori* infection, whereas the diffuse type is mainly hereditary and possesses multiple mutations [2, 3]. In the study of gastric cancer, epigenetics has been thought of as a critical factor in developing new strategies for the understanding of molecular processes [4]. The changes in the profile gene expression are the fundamental properties of aggressiveness and good tools in the prognosis of several oncological diseases [5]. New genes in the genomic context are attracting attention by playing essential roles in the progression of the disease [6].

The tight junctions (TJs) are intercellular adhesion complexes located in the apical zone of the lateral membrane of the epithelial and endothelial cells. TJs are essential for barrier function and cell polarity by limiting the movement of the proteins within the plasma membrane and regulating the paracellular solute [7]. TJs are made up of adapter proteins and integral membrane proteins, such as claudins, which are indispensable for TJ strand formation [8]. Claudins, a family of 27 isoforms, which, in addition to having barrier and polarity functions, also function as coreceptors for viruses and bacterial toxins, are coexpressed in specific cell types and are functionally divided into four groups with different functions [9]. The transcription of claudins is tightly controlled by regulatory mechanisms, epigenetic alterations, transcriptional changes, and post-translational modifications [10]. Nevertheless, the understanding of the structure and function of TJ has been amplified as the appearance of chimeric claudins [11]. The repercussion of other components, such as junctional adhesion molecules, zonula occludens proteins, membrane lipids, and mechanical forces, have transformed our knowledge of the structure, dynamic interaction, and function of TJ assembly [12]. Interestingly, the functional crosstalk between TJ proteins and signaling pathways involved in cell proliferation, transformation, and metastasis is becoming a current topic [13].

Claudins play a crucial role in regulating cellular processes such as growth, proliferation, and migration [14, 15]. The disruption of cell-cell junctions results in the loss of cell polarity and has a decisive role in cancer progression [16]. Claudin expression is altered, elevated, and negatively regulated in cancer, but most intriguingly, claudin expression is tumor-specific [17]. Bioinformatics analysis has extensively confirmed the prevalence of claudins in gastric cancer patients [18–22], especially Claudin-6 and -9, which are considered critical factors related to poor prognosis in gastric cancer [23, 24]. Claudin-9 is expressed primarily in the inner ear and is essential for hearing [25], however, its aberrant expression has been reportedly established in lung cancer [26], pituitary oncocytoma [27], and cervical carcinoma [28]. Claudin-9 has been included in a seven-gene signature as a clinical prognosis marker in esophageal adenocarcinoma [29]. Despite the above, there is a limited body of literature that recognizes the significance of Claudin-9 in gastric cancer.

The increase in Claudin-9 expression in the gastric cancer tumor tissue is associated with an increase in cell proliferation and invasion. Furthermore, Claudin-9 overexpression is associated with a higher mortality rate (66%) in diffuse-type gastric cancer [30, 31]. The present work aimed to perform a bioinformatics analysis to understand the mechanisms involved in the transcriptional and epigenetic regulation of Claudin-9 and its repercussions in gastric cancer.

## 2. Material and Methods

### 2.1. Claudin 9 (CLDN9) Datasets

*CLDN9* gene sequence was obtained from National Center for Biotechnology Information (NCBI, https://www.ncbi.nlm.nih.gov/) (Gene ID: 9080), and a blast was performed to obtain the reference number and the sequence in FASTA format. Subsequently, the Genome Browser database (https://genome.ucsc.edu/) was used to analyze the genomic context of the *CLDN9* gene. In the sequence section, upstream of the 5′ end was adjusted to 2500 bp for further promoter analysis.

### 2.2. Data Processing

Firstly, we look to determine the minimum promoter region using “*neural network promoter prediction*.” In addition, we evaluated the presence of consensus regulatory sequences, such as CpG islands, TATA, CAAT and GC boxes, GATA, Inr (Initiator), and DPE (downstream promoter element) sequences.

### 2.3. Prediction of Transcription Factors

TRANScription FACtor database (TRANSFACT; http://gene-regulation.com) was used to identify possible transcription factors and their binding sites in the *CLDN9* gene promoter. TRANSFACT uses two algorithms. The first describes the binding sites in the promoters or enhancers, and the second shows the transcription factors. Again, using a 2500 bp sequence upstream of the transcriptional start site of *CLDN9*, we perform the analysis. The search settings were established in the match: vertebrates, minimize the sum of both errors pattern: 0.7–0.75 cut-off—best selection-prf. A similarity of 100% was considered for further analysis of molecular docking with their corresponding promoter sequence.

We use the Signaling Pathway Project experimental database (https://www.signalingpathways.org/index.jsf), to study the possible transcription factors. The “*Ominer*” tool (https://www.signalingpathways.org/ominer/query.jsf) was used to discover the relationship between the single gene *CLDN9* and the node of transcription factors using cistromics datasets (Chip-Seq) in the bio sample category of human in the stomach tissue.

### 2.4. Molecular Docking Analyses

Crystal availability and biological relevance in the cancer context were considered for factor selection and molecular docking analysis. The crystallographic structures were obtained from the Protein Data Bank (http://www.rcsb.org/pdb). The *Homo sapiens* crystals used were OCT-1 (1E3O; 280–438 aa), NF-kappaB (1SVC; 2–365 aa), AP-1 (1JNM; 254–315 aa), C-ets-1 (1GVJ; 297–441 aa), and HNF-3*β* (5X07; 157–258 aa). Three-dimensional DNA models were built using “*The Supercomputing Facility for Bioinformatics & Computational Biology*,” IIT Delhi (SCFBio, http://www.scfbio-iitd.res.in/). Docking studies were calculated using different configurations of the HDOCK server (http://hdock.phys.hust.edu.cn/). Finally, the visualization of the molecular models was done using Chimera software ver. 1.11.2.

### 2.5. Methylation Bioinformatic Analyses

Disease meth version 2.0 database (http://bio-bigdata.hrbmu.edu.cn/diseasemeth/) concentrates the aberrant methylomes of different types of cancer. Methylation was analyzed from the Cancer Genome Atlas (TCGA) Gastric Cancer Patient Data Repository (STAD). The data were obtained using the Illumina Infinitum HumanMethylation 450 Beadchip technology experimental platform (*n* = 397). The differential analysis method was Student's *t*-test. *P*=0.05 was considered a significant value. The absolute methylation difference was set at >0.2. The methylation analysis of the promoter region (2 kB upstream to TSS to 0.5 kB downstream) of CLDN9 was performed using the MethBrowser tool.

Xena server (https://xena.ucsc.edu/) was used to explore the possible associations of the CLDN9 promoter methylation with its expression levels and differential methylation between the histological types. Likewise, the impact of CLDN9 methylation on different clinical consequences, such as overall survival (OS), progression-free interval (PFI), and the disease-free interval was analyzed.

### 2.6. Structural Characteristic Analyses

For the study of the structure, sequence, and domains of CLDN9, the polypeptide sequence was obtained using the databases Uniprot-KB (https://www.uniprot.org/), secondary structure prediction was determined using Expasy-SOPMA software (https://npsa-prabi.ibcp.fr/cgi-bin/npsa_automat.pl?page=/NPSA/npsa_sopma.html), protein data bank (https://www.rcsb.org/), proteomics DB (https://www.proteomicsdb.org/), and Interpro (https://www.ebi.ac.uk/interpro/). The FASTA protein sequence was used for all analyses. We chose the 095484 entries in UniprotKB, CLDN9_HUMAN entry name, *Homo sapiens* organism.

In the case of Expasy-SOPMA, we used UNK_645620 ID protein for Claudin-9 [32].

Pharos was used to analyze the data compiled in a knowledge management base taking into account the complexity of the analyzed targets. With this tool, it was sought to efficiently visualize and summarize the results to identify the patterns. The information reviewed and related to Claudin-9 was tissue and cell type, cell component, disease disturbance, genetic alterations, metabolism, molecular function, protein domains, PubMedID, SNP, and transcription factors, among others.

### 2.7. Protein-Protein Interactions Networks

The general interaction map of Claudin-9 was generated using the String database (https://string-db.org/cgi/input?sessionId=biUPDH3R9Of4&input) to analyze known interactions (from curated databases and experimentally determined), predicted interactions (gene neighborhood, gene fusions, and gene co-occurrence), and others (textmining, coexpression, and protein homology). In String, the lines denote the physical or theoretical interactions, and the ends of each line denote the effect on the protein. In this case, the circle at the end of the line indicates that the result is unknown. The analysis of interaction was performed using confidence fields. The thicker the line and the closer they are to each other, the greater the interaction confidence. Additionally, we used BioGRID (https://thebiogrid.org/) to investigate the physical interaction with Claudin-9. The settings used for the assays in both databases were CLDN9; primary external reference: UniProt O95484; species: *Homo sapiens*; Identifier: R-HSA-421271.

### 2.8. Expression and Survival Analyses

The clinical impact was evaluated using the Xena Functional Genomics Browser (https://xenabrowser.net/). In addition, data from 580 samples from the TCGA Stomach Cancer Study (STAD) were used to analyze the impact of Claudin-9 expression in gastric cancer patients.

For the analysis of Claudin-9 expression in the different histological types of gastric cancer (TCGA STAD study), we compared the histologic type and the gene expression by RNAseq-IlluminaHiSeq UNC for the CLDN9 gene. The statistical test to determine the differences in the expression of Claudin-9 in the histological types was one-way ANOVA.

The overall survival (OS) and progression-free interval (PFI) of the patients and their association with the expression of the CLDN9 gene generated by RNAseq-IlluminaHiSeq UNC were analyzed. For OS, the samples were divided into low- (< 3,679) (*n* = 222) and high- (≥ 3,679) (*n* = 221) CLDN9 expression. For the case of PFI, the samples were divided into low- (< 3,679) (*n* = 223) and high- (≥ 3,679) (*n* = 222) CLDN9 expression. All survival analyses were performed and analyzed using the Kaplan Meier plots.

## 3. Results

### 3.1. Prediction of Transcriptional Regulatory Elements and Promoter of CLDN9

**Figure 1:**
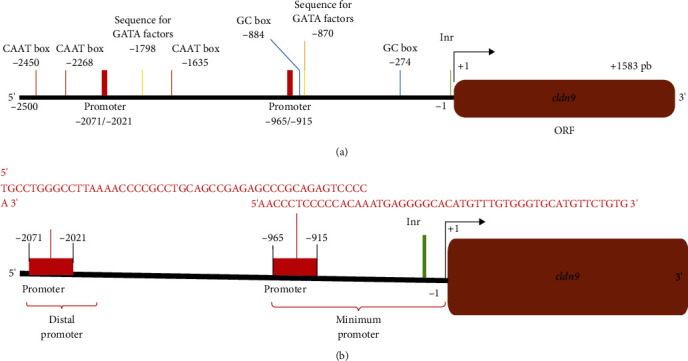
CLDN9 gene promoter region. (a) Elements of transcription regulation of the region 2.5 kB upstream (5′) to TSS to 0.5 kB downstream (3′). (b) Distal (score 1.00) and Minimun (score 0.89) promoter regions determined by Neural Network Promoter Prediction software.

The features of the CLDN9 gene promoter 2500 bp upstream of the transcription start point were screened. As shown in Figure 1(a), the promoter sequence has an unconventional structure as different transcriptional regulation elements are distributed throughout the 2500 bp. The Neural Network Promoter Prediction software analysis identified two sequences that interact with the GATA factors and GC and CAAT boxes. According to the conventional definition, one GATA binding sequence, two GC boxes, and the initiating region integrated a minimal promoter region. The transcription start point was located between −965 and −915 bp. A distal promoter was also detected between −2450 and −1635 bp. Three CAAT boxes integrated this promoter and one GATA binding sequence, with the transcription start point located between −2071 and −2021 bp (Figure 1(b)).

### 3.2. Transcription Factors

Twenty-two transcription factors were predicted by TRANSFACT to exist within the transcription start point and 2500 bp upstream (Table 1). The score of all of them is 100%, thus implying a high possibility of binding with the promoter sequence. Interestingly, most of them are strongly related to cancer initiation and progression.

**Figure 2:**
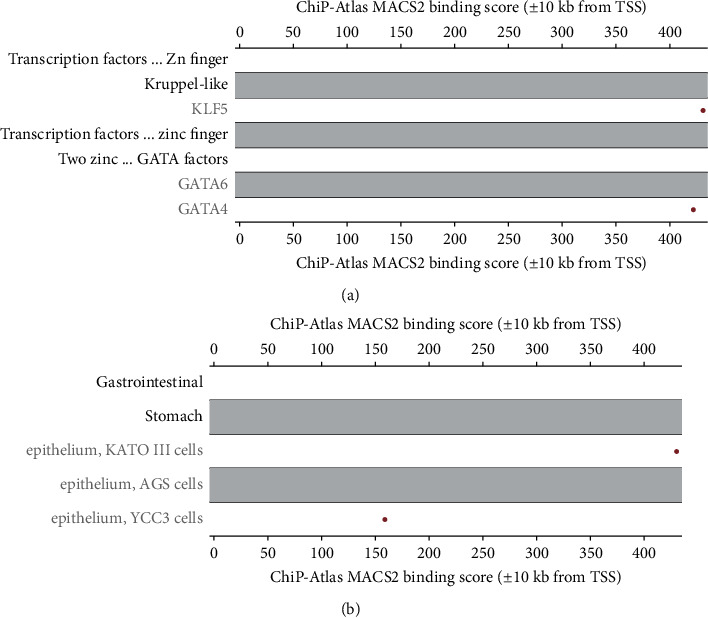
Transcription factors bound to the CLDN9 promoter region. (a) Binding score of transcription factors bound to the CLDN9 promoter region determined by ChiP assays (ChiP-Atlas MACS2) obtained from the Signaling Pathway Project. (b) Cell lines used to determine the transcription factors involved in the regulation of *cldn9* gene.

The analysis performed in the Signaling Pathway Project showed GATA6 and GATA4 as the regulators of CLDN9 expression in the YCC3 and AGS epithelial cell lines, respectively, whereas KLF5 was the regulator in KATO III cells (Figures 2(a) and 2(b)).

### 3.3. Molecular Docking Analysis

**Figure 3:**
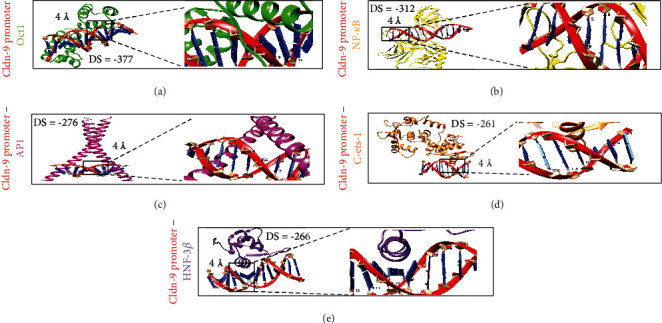
Molecular docking of the CLDN9 promoter region with transcription factors. Docking between the CLDN9 promoter (red/blue) and (a) Oct1 (green), (b) NF-kB (yellow), (c) AP1 (pink), (d) C-ets-1 (orange), and (e) HNF-3*β* (purple). Right side panels represent a zoom image of the interaction areas and the interacting nucleotides. DS = docking score.

Anchorage analysis was performed to predict the binding affinity of c-Ets-1, NF-*κ*B, HNF-3*β*, Oct1, and AP-1 to the CLDN9 promoter. The docking scores obtained were −377 for Oct1, −312 for NF-*κ*B, −276 for AP-1, −261 for c-Ets-1, and −266 for HNF-3*β* (Figure 3). All distances between the interacting residues were <4 Å.

### 3.4. Epigenetic Regulation Analysis

A comparison with the normal controls showed an increasing trend of methylation in the stomach cancer samples (Disease meth v. 2.0 STAD database). Therefore, a more exhaustive bioinformatics analysis of the promoter region was performed using the data from the cancer genome atlas.

**Figure 4:**
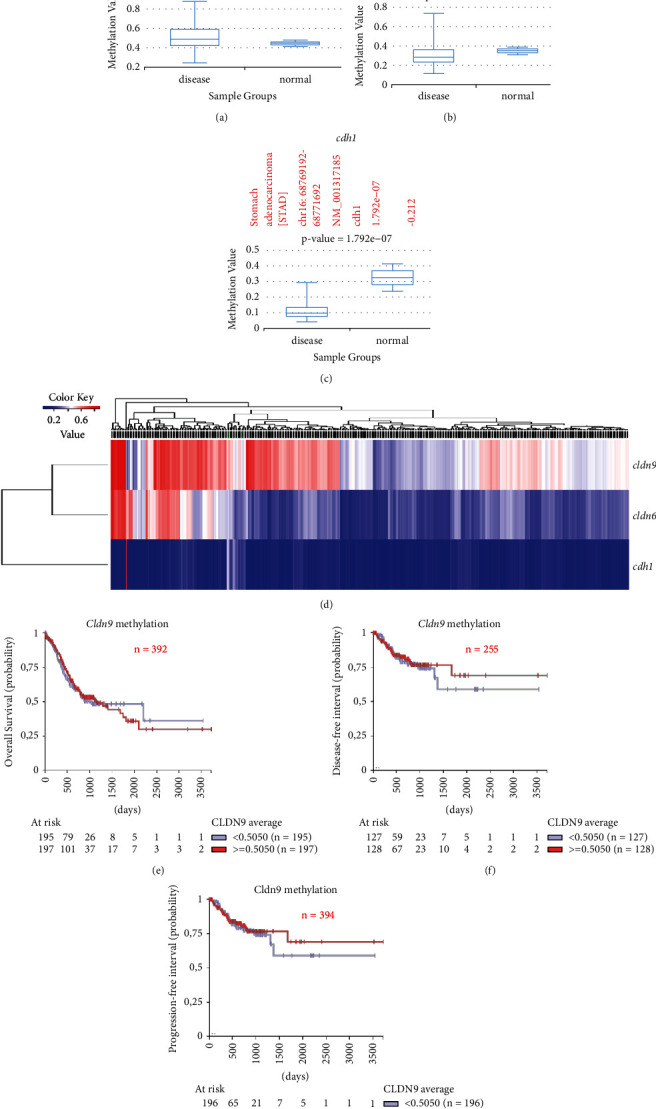
Methylation of CLDN9, CLDN6, and CDH1 genes. Methylation level of (a) CLDN9, (b) CLDN6, and (c) CDH1 under gastric cancer condition compared with normal tissue. (d) Heatmap of the methylation of CLDN9, CLDN6, and CDH1 genes. (e) Overall Survival probability between CLDN9 high-methylation level (≥0.5050 average) vs. low-methylation level (<0.5050 average), *P*=0.6871, Log-Rank = 0.162. (f) Disease-free interval between CLDN9 high-methylation level (≥0.5050 average) vs. low-methylation level (<0.5050 average), *P*=0.7238, Log-Rank = 0.125. (g) Progression-free interval between CLDN9 high-methylation level (≥0.5050 average) vs. low-methylation level (<0.5050 average), *P*=0.3450, Log-Rank = 0.892.

Because of the closeness in the genomic context and their functional similarities, a comparison of the promoter's methylation between the CLDN9 and CLDN6 genes was performed. Cadherin 1 gene, a gene regulated by methylation in the gastric tissue, was added as a control. The CLDN9 gene was highly methylated in contrast to CLDN6, which showed lower levels (Figures 4(a) and 4(b)). The methylation of CDH1 was significantly lower (Figure 4(c)). The heat map results of claudin gene methylation showed transcriptional repression of the CLDN9 gene expression in gastric cancer patients (Figure 4(d)).

High- and low-risk values for the CLDN9 gene were calculated according to the median value of gene methylation. There were no significant differences in overall survival between high- and low-methylation groups (*P*=0.6871) (Figure 4(e)). Similarly, the group with the highest methylation remained disease-free slightly longer without disease recurrence than the group with the lowest methylation (*P*=0.7238) (Figure 4(f)). The progression-free interval was slightly better but not significant in the lower methylation group (*P*=0.1249) (Figure 4(g)).

### 3.5. Secondary Structure of Claudin-9

**Figure 5:**
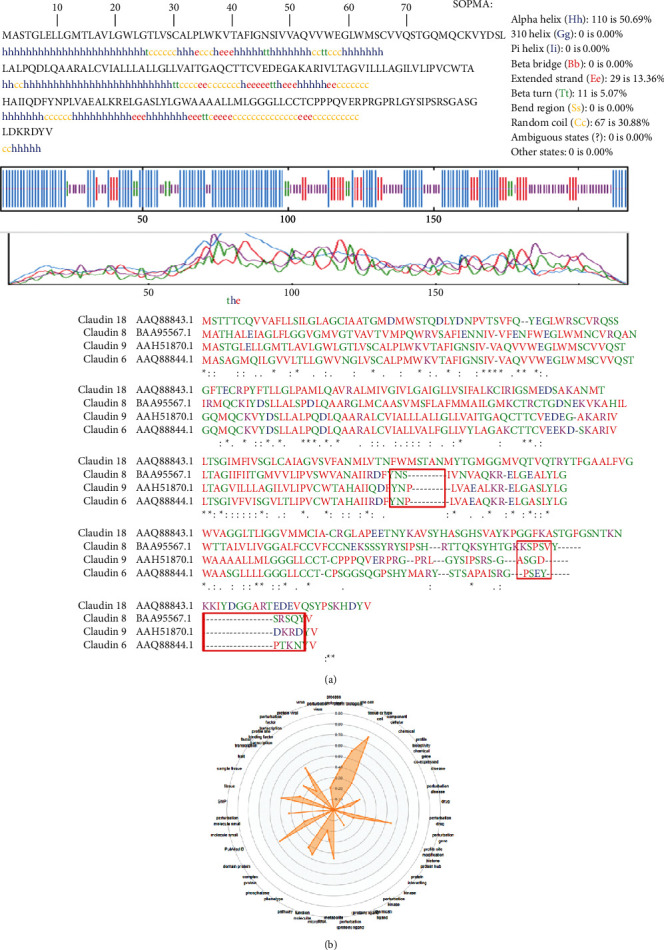
Sequence and structural analysis of Claudin-9. (a) Sequence comparison between Claudin-9 and those of other closely related claudins. The structural components of Claudin-9 are enlisted, and (b) the analysis of the knowledge of Claudin-9 based on its molecular components and characteristics, as well as its interactions.

The CLDN9 gene codifies an RNA that translates a protein with 217 amino acids of 22.8 kDa. The predicted secondary structure of Claudin-9 using the SOPMA method identified *α*-helixes (50.69% of the sequence), extended strands (13.36% of the sequence), *β*-turns (5.07% of the sequence), and random coils (30.88% of the sequence) (Figure 5(a)). Interpro databases were used to identify nonconserved (blue) and nonintegrated (brown and pistachio green) elements within the protein. The most representative figure is the secondary structure prediction in which the transmembrane *α*-helix (blue, spiral structure), sheet (red, folding), turn (green), and coil (purple) generate an irregular curly structure (Figure 5(a)).

The sequence alignment results showed that Claudin-9 is highly similar to Claudin-6 and -8 as they possess 217 to 220 residues compared to Claudin-18 that has 261 residues. The analysis showed that there are regions present in Claudin-18 (148–159, 234–258) that are not in Claudin-6, -8, and -9.

The radial graph (Figure 5(b)) shows that more progress has been made in the detection of the expression of Claudin-9 in the tissues and cell types (0.75) than in the studies of the alteration in its expression (0.45), mentions in Pubmed (0.59), and the analysis of its transcription factors (0.45). The values below 0.7 are considered to have a low abundance of knowledge in the area.

### 3.6. Protein-Protein Interactions

**Figure 6:**
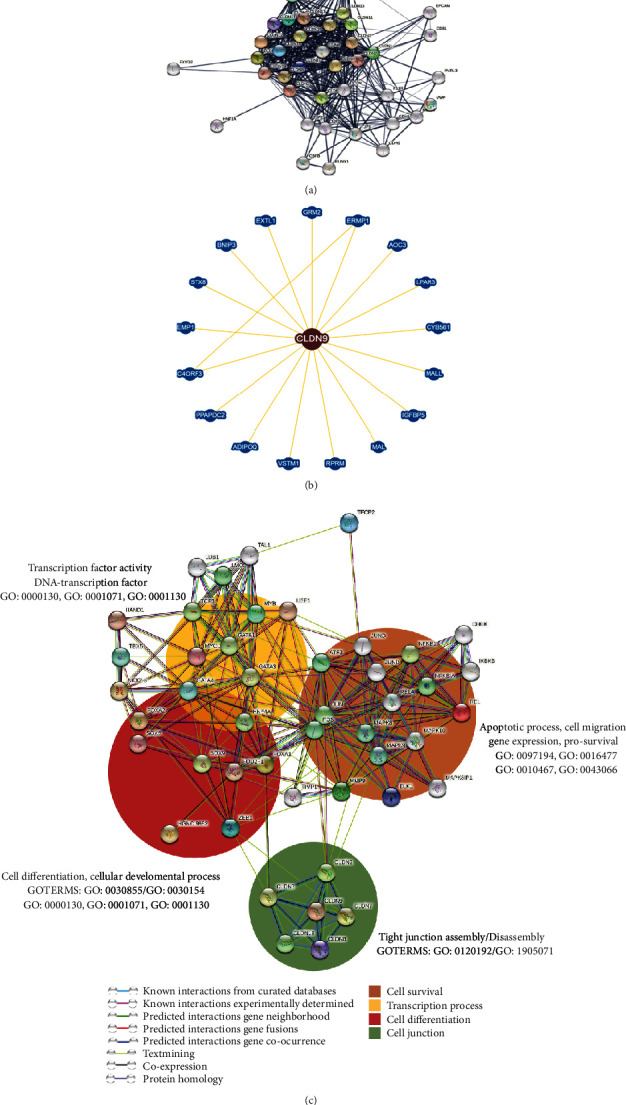
Protein-protein interaction network of Claudin-9. (a) The analysis of interaction by confidence using Reactome and (b) protein-protein interaction network by STRING. The lines denote physical or theoretical interactions, field 1 to 10, field 2 to 10 (the thicker the line and the closer they are to each other, the greater the interaction confidence), and (c) PPI interactions of Claudin-9 focused on related transcription factors. The background colored fields indicate functional affinity. The green field shows the functional affinity of the different claudins of the interaction network in the biological processes “Tight junction assembly/Disassmably (GOTERMS: GO: 0120192/GO: 1905071).” The red field shows the functional affinity of the transcription factors and other proteins participating in cell differentiation and the cell development process. The yellow field mainly shows the GATA transcription factors and other genes related to the development and progression of cancer, including gastric cancer.

The STRING interactome analysis showed that the first field of proteins with direct interaction or association with Claudin-9 is the claudin family proteins, such as Claudin-1, -2, -3, -8, -10, -12, -15, -16, -18, and -23, among others. In the second field, the proteins with indirect interaction with Claudin-9 are proteins, such as Cadherin-1 (CDH1), Cadherin-5 (CDH5), Occludin (OCLN), ZO1 (TJP1), ZO5 (TJP5), EPCAM, CD81, or JAM-A (F11R), among others. These proteins participate in the positive regulation of the blood-brain barrier permeability, calcium-independent cell-cell adhesion via plasma membrane, the establishment of the intestinal endothelial barrier, TJ assembly, and protein relocalization to the TJ (Figure 6(a)) (Table 2).

An analysis of the physical interaction network of Claudin-9 using the BioGRID database [72] showed 17 proteins that physically interact with Claudin-9 (Figure 6(b)). These proteins are not the typical interactors for Claudin-9, which points to its participation in still unknown processes. For instance, GRM2 and LPAR3 proteins are G protein-coupled receptors that regulate glutamate and lysophosphatidic acid uptake, respectively, or RPRM (Reprimo protein) that modulates the arrest of the p53-dependent cell cycle in the G2 phase.

Finally, an analysis of interactomes given by “textmining” between Claudin-9 and the transcription factors determined using TFSearch made it possible to determine a group of functions generally associated with four major cellular processes. (1) Cell junctions composed of other claudins. (2) Cell survival characterized by apoptotic or antiapoptotic processes, cell migration, and invasion processes; the most representative proteins involved in such processes were MAPK8, MAPK9, MAPK10, MAPK8IP1, and MMP9. (3) Cell differentiation and development, where the relevant proteins of these processes, such as the transcription factor Sox5 that participates in invasion and metastasis in gastric cancer and Sox9 that plays a role in gastric cancer development, were determined. Other important proteins in this group were the transcription factors FOXA1 and FOXA2 that have been reported as the initiating factors of a cellular transdifferentiation program that generates gastric-like tissue in lung adenocarcinomas. (4) Cellular transcription process, which included a great variety of transcription factors, such as USF1 that participates in familial combined hyperlipidemia, TCF3, which plays a significant role in B and T lymphopoiesis, or LMO2 that has a central role in erythropoiesis and hematopoietic development (Figure 6(c)).

### 3.7. Correlation with Survival in Gastric Cancer Patients

**Figure 7:**
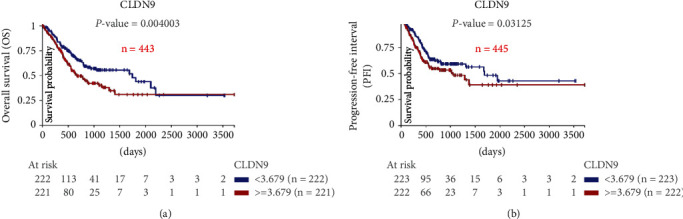
Expression of Claudin-9 and its clinical impact. (a) Claudin-9 expression and Overall Survival based on a high (≥3.679) vs. low (<3.679) expression, (b) Progression-free interval based on high (≥3.679) vs. low (<3.679) Claudin-9 expression.

The overall survival of patients, based on the survival data from “*The cancer genome Atlas (TCGA)*” from the STAD study (TCGA Stomach Cancer), shows a significant positive effect (*P*=0.004) in those patients with a lower expression of Claudin-9. Their median survival was 4.7 years compared to 1.6 years in the group with the highest expression (Figure 7(a)).

The progression-free interval showed that patients with higher Claudin-9 expression have a worse prognosis, presenting disease progression at a median of 2.7 years compared to the 4.5 years observed for the low-expression group (Figure 7(b)).

## 4. Discussion

Claudins conform to a family of proteins with 27 human isoforms that play a crucial role in the regulation of cellular processes, such as growth, proliferation, migration, and invasion [14]. Most members of the claudin family share the same intracellular framework, however, the difference between them lies in their extracellular domains that regulate gate, barrier, and coreceptor diversity [7, 9, 12, 73]. The CLDN9 gene has four introns and is located close to the CLDN6 gene in chromosome 16 [73]. It is considered, similar to CLDN6, a developmental claudin isoform [74]. Claudin-9 is expressed in the inner ear [25], where it acts as a cation barrier [75], a process essential for hearing, and it is also a coreceptor for hepatitis C virus and *C. perfringes* enterotoxin [76, 77]. Its RNA is poorly expressed in the stomach (0.3 average protein transcripts per million) although the protein level is not necessarily correlated with the mRNA level (The Human Protein Atlas) [78]. Claudin-9 is related to poor prognosis in gastric cancer [23, 30, 31]. Nevertheless, the prominence of Claudin-9 in relation to its interactions, activation, and role in gastric cancer is poorly described. Claudin-9 expression is related to the increased metastatic ability of the hepatocytes by disturbing the TyK2/Stat3 signaling pathway [79], and it has been related to lymphatic metastasis in cervical carcinoma [28].

The results showed that the CLDN9 promoter possesses a minimal promoter region integrated by one GATA binding sequence, two GC boxes, the initiating region, and a distal promoter integrated by three CAAT boxes and two GATA binding sequences. The GC box sequences are considered the common transcriptional regulatory elements. The CAAT boxes signal the binding site for general transcription factors, and the sequences for the GATA factors serve as controllers for activating or repressing transcription [80]. It is possible that the function of the translated protein, cell proliferation and/or cell apoptosis, might be dependent on which promoter region binds a given transcription factor or its binding kinetics [81]. It is also possible that the binding of both promoter regions by the same transcription factor by a mediator and a modification complex is required to form a loop that initiates protein transcription [82]. It has been established that a change in the transcription factor activity dependent on the transcription factor concentration alters the expression of its targets [83].

Interestingly, 20 different transcription factors that can bind the CLDN9 promoter region with a 100% score were determined. The majority are considered the key regulators of epithelial differentiation and organ development, i.e., CP2 [84], the maintenance of the germline stem cells, i.e., USF [85], enhancers that activate transcriptional programs and cellular reprogramming, i.e., GATA1 and GATA 3 [86], favor cell growth and proliferation, i.e., AP1 and AP4 [87], and facilitate TJ formation in carcinoma cells, i.e., HNF4 [88].

ChiP-Atlas MACS2 analysis found that GATA6 and GATA4 regulate CLDN9 expression in the YCC3 and AGS gastric epithelial cancer cell lines, respectively, whereas KLF5, a transcription factor that binds to the GC boxes [89] and interacts with GATA4 and GATA6 [90] was the regulator in KATO III gastric cancer cells. It is possible that the difference may arise from the different histopathological phenotypes because KATO III is a human gastric signet ring cell adenoma cancer that can be induced to adipogenic, chondrogenic, osteogenic, and neurogenic differentiation [91].

A different analysis showed that the CLDN9 gene was highly methylated and the heat map results showed the transcriptional repression of the CLDN9 gene expression in gastric cancer patients. DNA methylation is an epigenetic mechanism recognized as a biological process that can change the activity of a DNA segment, silencing gene expression [92]. Some genes can be expressed even when they are extensively methylated [93, 94]. In relation to gastric cancer, the hypermethylation of the Claudin-11 promoter has been associated with increased invasive potential [95], and the hypermethylation of the Claudin-3 promoter is considered a predictor of poor prognosis in advanced gastric adenocarcinoma [96]. In fact, promoter hypermethylation and claudin expression have been associated with gastric cancer TNM stage [97, 98].

As expected, Claudin-9 expression is associated with shorter and progression-free survival. The expression of dedifferentiation markers, such as CD44, CD133, Claudin-6 or -9, in the majority of epithelial cancers [99–103] is associated with poor survival.

## 5. Conclusion

Claudin-9 is a TJ protein involved in key biological processes. Its expression may be regulated by many mechanisms, however, its overexpression or the methylation status of the promoter may be a prognostic factor in gastric cancer.

## Figures and Tables

**Table 1:** Transcription factors.

Transcription factors	Score	Strand position	Sequence
ZID	100	61 (+)	gGGCTCcagcaca
CP2	100	69 (+)	gcacacCCCAG
c-Ets-1	100	284 (−)	tgccTCCGGT
Nkx2-5	100	464 (+)	tcAAGTG
Elk-1	100	602 (−)	aaccTTCCGattta
USF	100	714 (−)	acctCGTGAa
MyoD	100	771 (−)	ccgcACCTgcc
AREB6	100	771 (+)	ccgcACCTgcc
SOX-9	100	799 (+)	agacaACAATcctc
Sox-5	100	801 (+)	acaACAATcc
HNF-4	100	924 (−)	agtgggtCTTTGaccaaag
c-Rel	100	1055 (+)	gggttTTTCC
GATA-3	100	1318 (+)	ggaGATCTta
v-Myb	100	1412 (−)	ggCCGTTgcc
RFX1	100	1414 (−)	ccGTTGCcagggcgatgc
NF-kB	100	1479 (+)	cgGGGACcttcccc
HNF-3*β*	100	1586 (+)	gggcaTGTTTgcatc
Oct-1	100	1589 (−)	catgTTTGCatcacg
AP-4	100	1624 (+)	agaggCAGCTggggaggg
GATA-1	100	1919 (−)	tccccTATCCcggc
E47	100	2082 (+)	cagtgcgTCTGGaggg
AP-1	100	2391 (+)	ctgAGTCAc

**Table 2:** STRING interactome.

Protein	Function	Type of interaction	Curated	References
EPCAM	The epithelial cell adhesion molecule's precursor participates in migration, proliferation, and cellular differentiation. In cancer, it promotes tumor progression by the EGFR pathway.	Textmining	STRING	[33, 34]

CD81	The tetraspanin family is a cell-surface protein and plays an essential role in developing cellular growth and activating the B and T cells. It is expressed in most types of cancers.	Textmining	STRING	[35, 36]

PVRL3	Poliovirus receptor-related 3, also called Nectin-3, forms part of the adherens junctions. PVLR3 has been implicated in tumor progression in lung, pancreatic, and ovarian adenocarcinomas.	Textmining	STRING	[37–39]

VWF	Von Willebrand factor, its primary role is in thrombus formation and blood coagulation. VWF has been reported to participate in inflammation, angiogenesis, and metastasis in various cancers, including gastric cancer.	Textmining	STRING	[40, 41]

F11R	Junctional adhesion molecule A (JAM-A) plays a role in the epithelial tight junction formation. JAM-A has been reported to promote proliferation and inhibit apoptosis in gastric cancer.	Textmining	STRING	[42, 43]

CDH5	Cadherin-5, this protein plays a role in endothelial adherens junction assembly and maintenance. In cancer, it has been reported to be involved in progression and metastasis. In gastric cancer, its expression correlates with poor survival.	Textmining	STRING	[44–46]

TJP1, TJP2, TJP3	Tight junction proteins ZO-1, ZO-2, and ZO-3 are closely related scaffolding proteins that link the tight junction (TJ) transmembrane proteins, such as claudins, junctional adhesion molecules, and occludin to the actin cytoskeleton. Their expression is altered during tumor development, metastasis, and poor prognosis in several types of cancers.	Texmining	STRING	[47–52]

OCLN	Occludin may play a role in forming and regulating the tight junction (TJ) paracellular permeability barrier. In gastric cancer, the downregulation of some occludins is associated with tumor aggressiveness and survival.	Texmining	STRING	[53, 54]

RUNX1	Runt-related transcription factor 1 is a transcription factor that modulates the target genes involved in the development of immune cells. In colorectal cancer, it promotes tumor metastasis by activating the Wnt/*β*-catenin signaling pathway and EMT.	Textmining	STRING	[55–57]

CBFB	Core-binding factor subunit beta, a non-DNA-binding regulatory subunit that allosterically enhances the sequence-specific DNA-binding capacity of RUNX—involved in hematopoiesis and osteogenesis. In breast cancer, it has been observed as a tumor suppressor.	Textmining	STRING	[58, 59]

HNF1A	Hepatocyte nuclear factor 1-alpha is a transcriptional activator that regulates the tissue-specific expression of multiple genes, especially in the pancreatic islet cells and the liver. Recently, it has been reported to facilitate gastric cancer tumor progression.	Textmining	STRING	[60, 61]

FXYD2	Sodium/potassium-transporting ATPase subunit gamma may form the receptor site for cardiac glycoside binding or modulating the sodium ATPase's transport function. It may be involved in tumor growth.	Textmining	STRING	[62, 63]

CAPN9	Calpain-9 is involved in apoptosis, cellular proliferation, and cell motility. Calpain expression is altered during tumorigenesis and the proteolysis of numerous substrates, such as inhibitors of nuclear factor-*κ*B and proto-oncogenes.	Texmining	STRING	[64–66]

MFHAS1	Malignant fibrous histiocytoma-amplified sequence 1 functions in innate immunity, more specifically, in the inflammatory response as a regulator of the Toll-like receptor TLR2 and TLR4 signaling pathways. MFHAS1 promotes colorectal cancer progress by regulating the polarization of tumor-associated macrophages via the STAT6 signaling pathway	Textmining	STRING	[67, 68]

PPP1R3B	Protein phosphatase 1 regulatory subunit 3B acts as a glycogen-targeting subunit for phosphatase PP1 and promotes glycogen synthesis. In cancer, it has been used to treat a melanoma patient with an immunological focus.	Textmining	STRING	[69, 70]

ESAM	Endothelial cell-selective adhesion molecule's *in vitro* functional profile strongly suggests a role in cell-cell interactions critical for vascular development or function.	Textmining	STRING	[71]

## Data Availability

The data used to support the findings of this study are available from the corresponding author upon request.
